# A Survey on Machine Learning and Internet of Medical Things-Based Approaches for Handling COVID-19: Meta-Analysis

**DOI:** 10.3389/fpubh.2022.869238

**Published:** 2022-06-23

**Authors:** Shahab S. Band, Sina Ardabili, Atefeh Yarahmadi, Bahareh Pahlevanzadeh, Adiqa Kausar Kiani, Amin Beheshti, Hamid Alinejad-Rokny, Iman Dehzangi, Arthur Chang, Amir Mosavi, Massoud Moslehpour

**Affiliations:** ^1^Future Technology Research Center, College of Future, National Yunlin University of Science and Technology, Douliou, Taiwan; ^2^Department of Informatics, J. Selye University, Komárom, Slovakia; ^3^Department of Design and System Operations, Regional Information Center for Science and Technology (R.I.C.E.S.T.), Shiraz, Iran; ^4^Department of Computing, Macquarie University, Sydney, NSW, Australia; ^5^BioMedical Machine Learning Lab, The Graduate School of Biomedical Engineering, U.N.S.W. Sydney, Sydney, NSW, Australia; ^6^U.N.S.W. Data Science Hub, The University of New South Wales (U.N.S.W. Sydney), Sydney, NSW, Australia; ^7^Health Data Analytics Program, AI-enabled Processes (A.I.P.) Research Centre, Macquarie University, Sydney, NSW, Australia; ^8^Department of Computer Science, Rutgers University, Camden, NJ, United States; ^9^Center for Computational and Integrative Biology, Rutgers University, Camden, NJ, United States; ^10^Bachelor Program in Interdisciplinary Studies, National Yunlin University of Science and Technology, Douliu, Taiwan; ^11^John von Neumann Faculty of Informatics, Obuda University, Budapest, Hungary; ^12^Institute of Information Engineering, Automation and Mathematics, Slovak University of Technology in Bratislava, Bratislava, Slovakia; ^13^Department of Business Administration, College of Management, Asia University, Taichung, Taiwan; ^14^Department of Management, California State University, San Bernardino, CA, United States

**Keywords:** machine learning, COVID-19, Internet of Things (IoT), deep learning, big data, information systems, internet of medical things, coronavirus

## Abstract

Early diagnosis, prioritization, screening, clustering, and tracking of patients with COVID-19, and production of drugs and vaccines are some of the applications that have made it necessary to use a new style of technology to involve, manage, and deal with this epidemic. Strategies backed by artificial intelligence (A.I.) and the Internet of Things (IoT) have been undeniably effective to understand how the virus works and prevent it from spreading. Accordingly, the main aim of this survey is to critically review the ML, IoT, and the integration of IoT and ML-based techniques in the applications related to COVID-19, from the diagnosis of the disease to the prediction of its outbreak. According to the main findings, IoT provided a prompt and efficient approach to tracking the disease spread. On the other hand, most of the studies developed by ML-based techniques aimed at the detection and handling of challenges associated with the COVID-19 pandemic. Among different approaches, Convolutional Neural Network (CNN), Support Vector Machine, Genetic CNN, and pre-trained CNN, followed by ResNet have demonstrated the best performances compared to other methods.

## Introduction

The outbreak of COVID-19 in Wuhan City, Hubei Province, China, began in December 2019 through the seafood wholesale market (1). Later, on January 30, 2020, the World Health Organization (WHO) declared the prevalence of Covid-19 as an emergency pandemic worldwide (2). Many governments have declared it a dangerous pandemic and imposed full quarantine to prevent the spread of COVID-19. Several countries have reduced their growing infection by tightening quarantine and forcing people to maintain social distance (3). Even if through complete quarantine, they failed to control the COVID-19 completely. Some countries have joined in the medical development to treat COVID-19. However, to date, there is no specific drug to treat COVID-19. However, few drugs have been suggested as potential research therapies. The proposed drug has been studied under WHO-led clinical trials (4). According to several studies, since COVID-19 is a communicable disease, the WHO has stated that complete quarantine could be the only way to prevent COVID-19 (5).

The COVID-19 outbreak has created many challenges in human life worldwide (6). The most devastating impact, increasing casualties and deaths (around the world), has made it clear the need for social and business restrictions (7). With the expansion of the COVID-19 pandemic, the world community has faced many other problems in various aspects of life, such as economic and social life, psychological wellness, political interactions, cultural activities, educational limitations, religious restrictions, and even sports events (8, 9). Such examples highlight the need for effective and intelligent systems to deal with such crises in the pandemic situation (9). Early diagnosis, prioritization, screening, clustering and tracking of patients, and production of drugs and vaccines are some of the applications that have made it necessary to use a new style of technology to involve, manage, and deal with this epidemic (10). Machine Learning (ML) and Artificial intelligence (AI) algorithms displayed promising ability in prediction and classification (11–22) including disease prediction (23–34), virus genome analysis (24, 35, 36), and medical imaging and Internet of Things (37–40). Strategies backed by artificial intelligence (A.I.) and the Internet of Things (IoT) have been undeniable to understand how the virus works and try to prevent it from spreading (9, 41). These techniques have evolved with the development of computing resources with cloud computing and recent advances in ML. These advances enable researchers to process large amounts of data and extract information. ML-based methods used in processing and modeling data on COVID-19 disease can increase efficiency and speed up results by improving computations. Several researchers have moved toward using ML-based techniques for different applications in the COVID-19 dataset, such as classification using C.T. Images (42), chest C.T. Images (43), and X-ray images (44).

Given the diversity of data, applications, and even the multiplicity of machine learning methods, it is necessary to develop a comprehensive survey study that can consider all the strengths and weaknesses in a standard and systematic study. Table 1 presents similar survey studies developed in the field for describing their ability to convey their message on the subject reviewed. Table 1 discusses the study's strengths to find the main research gap.

**Table 1:** The description of the conducted review articles.

**Reference**	**Highlights**	**Database information**	**Probable gap**
Guo et al. (45)	ML for COVID-19 Diagnosis	NA.	Limited field of the study and lack of proper database information
Abumalloh et al. (46)	ML methods for processing the medical image in the context of the COVID-19 crisis	Eight electronic databases: Elsevier, IEEE, PubMed, Wiley Online Library, Springer, Summon, Google Scholar, and Taylor and Francis	Limited field of the study and proper evaluation
Khan et al. (47)	AI for preventing the COVID-19 pandemic	ScienceDirect, Google Scholar, and preprints from arXiv, medRxiv, and bioRxiv	Subject review interval and evaluation of methods
El-Rashidy et al. (48)	The role of A.I. in preventing the COVID-19 pandemic	Textual data, medical images, and speech data	The subject review interval
Alballa and Al-Turaiki (49)	ML techniques for COVID-19 diagnosis, mortality, and violence risk estimation	PubMed, Scopus, IEEE Xplore, and Google Scholar	Limited subject review interval

In one of the early studies, Gou et al. presented a survey to evaluate the ML-based techniques for diagnosing COVID-19 using medical data collection, image preprocessing, feature extraction, and image classification. The study evaluates Transfer, ensemble, unsupervised and semi-supervised learnings, convolutional neural networks, graph neural networks, and explainable deep neural networks. Evaluations focused on the advantages and limitations of the diagnosis techniques (45). Abumalloh et al. presented a state-of-the-art ML-based technique for handling medical image processing in the context of the COVID-19 crisis (46). Khan et al. developed a survey of the applications of A.I. for preventing the COVID-19 pandemic (47). El-Rashidy et al. conducted a review study to describe A.I.'s role in preventing the COVID-19 pandemic using the five applications, including COVID-19 diagnosis, estimation of the COVID-19 outbreak, and patient characteristics, as well as vaccine development (48). Later on, Alballa and Turaiki surveyed the recent articles on ML techniques for COVID-19 diagnosis, mortality rate prediction, and violence risk estimation (49). As can be deduced, many survey studies have been developed. But, the existence of a study that can systematically review and discuss two interrelated areas of the ML and the IoT in the form of an article has been lost from the research literature.

The main contribution of this study is to systematically investigate and analyze the role of ML and the Internet of Medical Things (IoMT) to address the challenges associated with diagnosis of the COVID-19 and its outbreak prediction. Here we comprehensively investigate the merits and shortcomings of the ML and IoMT tools proposed for these tasks and present a numerical and statistical analysis.

There is an urgent need to utilize existing technologies to their full potential. Internet of Things (IoT) and ML is regarded as one of the most trending technologies with great potential in fighting against the coronavirus outbreak. The IoT comprises a scarce network in which the IoT devices sense the environment and send valuable data on the internet. In this review, we examine the current status of IoT applications and ML related to COVID-19, identify their deployment and operational challenges, and suggest possible opportunities to contain the pandemic further.

The IoT provides the materials needed to help the world minimize the effects of COVID-19. The Internet of Things works with a wide range of applications to ensure compliance with health authorities' safety instructions and precautions. The Internet of Things has a scalable network with the potential to deal with the vast amount of data received from sensors used by several programs to combat COVID-19. In addition, reliable IoT networks reduce critical data delivery times, which can help provide a timely response during the global COVID-19 epidemic. Due to the prevalence of the COVID-19, the role of the Internet of Things was never as needed as it is now.

Artificial intelligence (A.I.) is one of the most important and promising technologies that help revolutionize many fields by creating a revolution. The introduction of machine learning algorithms and artificial intelligence to the Internet of Things has opened new doors in this field. Machine learning provides the opportunity to learn and extract meaningful patterns from data. Because IoT device data is collected in a database, it can easily be used to predict the prevalence and effects of the coronavirus and how to reduce it. Data of patients with COVID-19 help predict the future behavior of the virus and regional comparison of its effects. In addition, it also helps with the possible adaptation of COVID-19 symptoms to an effective and rapid A.I. treatment.

The patient's medical record and the results obtained help to predict better treatment choices based on artificial intelligence and machine learning (ML) algorithms and lead to rapid recovery and patient monitoring. Artificial intelligence-based emergency traffic control paves the way for ambulances and other emergency service providers. BlueDot was one of the first artificial intelligence companies to predict the outbreak of the Corona virus and identify its global threat. They provided information on the mobility pattern of the virus and its potential for spread. Other A.I. companies also joined hands to work with COVID-19, including Deargen, Insilico Medicine, and S.R.I. Biosciences and Iktos, Benevolent AI, DeepMind, Nanox, Baidu, Alibaba, and EndoAngel Medical Technology Co.

Here we conclude that there is a gap in how to address the strengths and weaknesses of machine learning and IoT methods that need to be addressed. In the meantime, to close this gap, we will need to classify, determine the pros and cons, challenges and limitations, and outline ways to deal effectively with COVID-19. In line with this basic need to have a deeper insight into the applications and effects of machine learning and the Internet of Things on the COVID-19 Pandemic, we presented research to be able to study these methods in different ways and in a practical way.

Accordingly, the main purpose of this review article is to examine the ML, IoT, and the integration of IoT and ML-based techniques in the applications related to COVID-19 from the diagnosis of the disease to the prediction of its outbreak.

The study has three main sections:

- A section for describing the studies developed by IoT and IoT-ML based techniques in COVID-19 applications.
- A section for presenting the role of ML-based techniques in COVID-19 applications.
- A section for presenting the main findings, challenges, and future perspectives.

## Methodology

### Dataset Preparation Method

A systematic review may provide technical and practical literature for a specific topic (50). A systematic review requires a proper collection of papers on the subject. Preparing a dataset is one of the main steps in determining review work quality (50). According to the P.R.I.S.M.A. guidelines, the present study has collected the most relevant studies from W.O.S. and Scopus libraries (51). There are four steps for preparing the database using the P.R.I.S.M.A. guidelines, including (52): (1) identification, (2) screening, (3) eligibility, and (4) inclusion. In the identification step, we employed the frequently used keywords, including COVID-19, pandemic, diagnosis, detection, Prediction, Monitoring, Classification, Identification, IoT, and Machine learning to search within the article title abstract and keywords. In the first step, about 109 articles have been identified and selected from the Thomson Reuters Web-of-Science (WoS) and Elsevier Scopus. In the Screening step, the duplicate articles have been removed. Twenty-five articles (about 23%) have been removed from the dataset. Thirty nine articles (about 36%) have been eliminated due to the lack of details on the methods, datasets, full text, etc. A total of 45 cases (about 41%) have been included in the screening step. In the eligibility step, the authors team surveyed the full text of the papers, and marked the relevant articles during monitoring eligibility. In this phase, 23 cases (about 22%) have been selected for investigating the evaluation criteria and including in the dataset.

### IoT for COVID-19

IoT is an interconnected set of computing tools from simple to complex that can be used in conjunction with mechanical or digital machines in the presence of humans, animals, or objects. IoT technology can easily transfer data from the source to the destination through the network without the operator's presence. This technology can be considered a special tool in human-human interaction or human-computer interaction (53, 54). An IoT platform includes the minimum equipment required, such as smart devices equipped with the web (55). These systems consist of processors, sensors, and communication hardware to collect, send, control, manage, and convert data into accessible data (55, 56). These systems connect to an IoT port used to send data to the cloud so that data can be analyzed and shared (57). These devices can operate by connecting to other related systems based on their information (58). These tools perform many of their tasks without human intervention.

**Figure 1:**
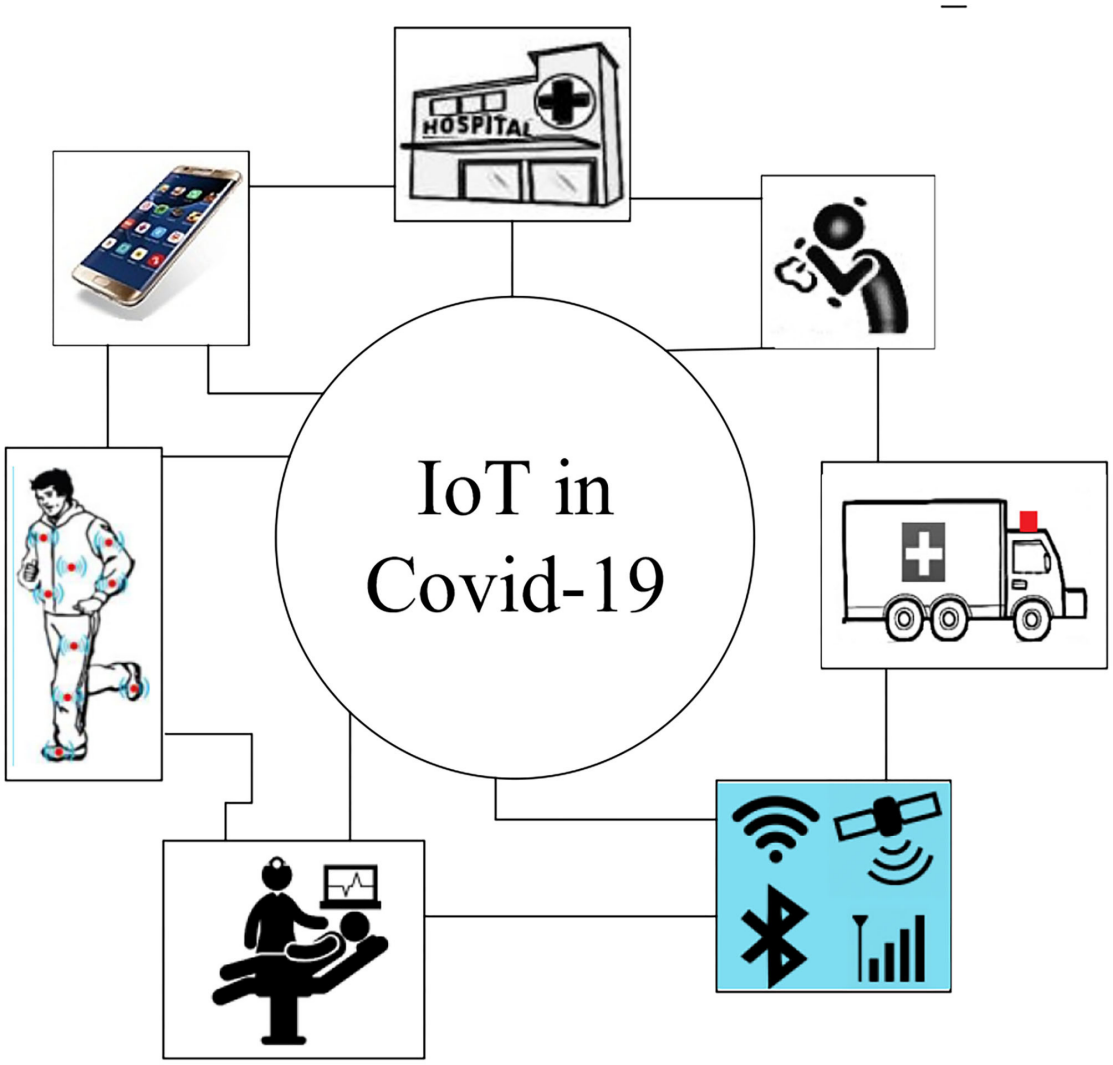
Applications of IoT in COVID-19.

Today, IoT technology in health and treatment is growing rapidly (59). The main applications of IoT in the process of treatment and intelligent health can include identification, digitization of medical information, patient transfer to the hospital, use of vital signs sensors, use of smartphones in communication, and digitization of medical processes (60, 61). Furthermore, IoT has become more popular and important due to the COVID-19 pandemic (62). Because this virus is highly contagious and has a high risk to human health, and has caused many problems for the medical staff, using non-contact methods to diagnose as soon as possible, control patients, monitor the condition of patients with acute illness, as well as maintain social distance, can be an important factor in breaking off part of the virus infection cycle (63, 64). In non-contact methods, the IoT is a leader and can solve many problems in this field (65). Figure 1 presents the main applications of the IoT in COVID-19 era.

Table 2 presents the highlighted studies for applying IoT-based techniques to tackle COVID-19. This table represents the studies based on the main four columns. First, the objective briefly describes the main objective of each study. Methodology/proposed algorithm presents the main algorithm and procedure employed by each study. Keyword indicates the main points and concentration of the study and finally, the application section presents the field of the application of each method.

**Table 2:** The main studies for the application of IoT based techniques for handling COVID-19.

**Order**	**Objective**	**Methodology/ Proposed Algorithms**	**Dataset**	**Keywords**	**Application**	**Reference**
1	To aim an innovative IoT-based online solution for tracking COVID-19 outbreaks	IoT-based platform to contact and to trace the infection	5G wireless, cloud technologies, and largescale data	I.O.T.: symptom-based device-to-device (D2D) communication	Prediction and monitoring	(66)
2	To compare DL techniques to detect COVID-19	DL-based COVID-19 diagnosis technique in order to model instances for each type and to diagnosis the vulnerabilities	Data from medical IoT devices	IOT: DL algorithm, AE	Diagnostic	(67)
3	To develop an IoT-based DL platform for early detection of COVID-19	Chest X-Ray pictures for training and testing of Regional-based Convolutional Neural Networks (R.C.N.N.) through IoT-based framework	Chest X-Ray images	IoT, COVID-19, Deep learning, Region Proposal Network (RPN)	Diagnostic	(68)
4	To develop a monitoring and detection system according to real-time data from in the presence of the machine learning algorithms	SVM, ANN, Naïve Bayes, K-NN, DT, Decision Stump, 1-R, and 0-R.	Actual COVID-19 patient identifiers include: Fever, Cough, Fatigue, Sore Throat, and Shortness of Breath	Machine learning algorithms, COVID-19	Identification and monitoring	(69)
5	To investigate the IoT for diagnosis of COVID-19 patients using interconnected network	12 IoT based monitoring systems are identified and discussed.	Dataset from databases of Google Scholar, PubMed, S.C.O.P.U.S. and ResearchGate	Internet of things (IoT)	Monitoring	(70)
6	To investigate participants' health conditions and remembering the maintain physical distancing	A lightweight and low-cost IoT node using a smartphone, and fog-based ML for data handling	Vital data from participations	Internet of Things (IoT), smartphone application, Machine Learning (ML), Fuzzy system	Monitoring	(71)
7	To aim a smart edge monitoring system using smart gadgets	To diagnose coronavirus infection using gadgets, deep edge computing and IoT to detect the virus suspected H2H chain	Data from sensors	COVID-19, Edge Computing, IoT	Monitoring	(72)
8	To employ a non-contact I.R. sensor to evaluate for the body temperature	Checking the health condition	Body temperature	IoT, detection system	Detection	(73)
9	To develop a Medical Diagnosis Humanoid to provide a complete diagnostic system for COVID-19	Autonomous navigation, detection, and monitoring system	Data from six different health modules	IoT, A.I., ML, Medical Diagnosis Humanoid	Monitoring and Diagnosis	(74)
10	To develop a low-cost robotic system to diagnosis and help virus affected people	To track hand gestures using radio frequency	Hand gesture	Wireless Robot, Gesture Recognition, IoT	Diagnosis and monitoring system	(75)
11	To contribute IoT and associated sensor technologies to trace, track and mitigate COVID-19 virus by developing hardware sensor	to integrate IoT techniques and provide insight on the expected outcomes	Temperature, Location, Imaging, Pay-point data, and Social media feeds dataset	A.I., IoT, big data, data sharing, cloud computing	Diagnosis and monitoring system	(76)
12	To extract the social relationships between mobile devices by allocating the limited protective resources	To employ dynamic W.U.G. model using social IoT	Pair of real-life datasets	Social Internet of Things; susceptible-exposed-infected-removed; reinforcement learning	Detection	(77)
13	To develop Internet of Things to prevent the spreading of COVID-19	Investigating an infected person using IoT	NA.	Internet of Things; health care; cloud computing	Detecting and Monitoring	(78)
14	To develop a platform for biometric face detection along with COVID-19 outbreaks	IoT-based Multi-task Cascaded Convolutional Network	Face image dataset	Detection, cascaded CNN, cloud computing, IoT, edge computing,	Detection and recognition	(79)
15	To introduce a high resolution A.Q. monitoring system	A preliminary validation of the Air Heritage pervasive Air Quality monitoring concept	Air quality dataset	Smart Air Quality monitors, IoT, Artificial Intelligence, COVID-19,	Monitoring	(80)
16	To develop an IoMT architecture with respect to combat COVID-19.	IoMT platform, emerging IoMT applications, to apply within the medical environment	N.A.	COVID-19, IoMT application, security	Detection	(38)
17	To test information technology for handling the COVID-19 pandemic	A.I., block-chain, Big Data and robots, for optimally handling pandemics	Google Scholar database and Proquest	COVID-19, information technology, A.I., big data, indonesia	Detection and monitoring pandemic	(81)

**Figure 2:**
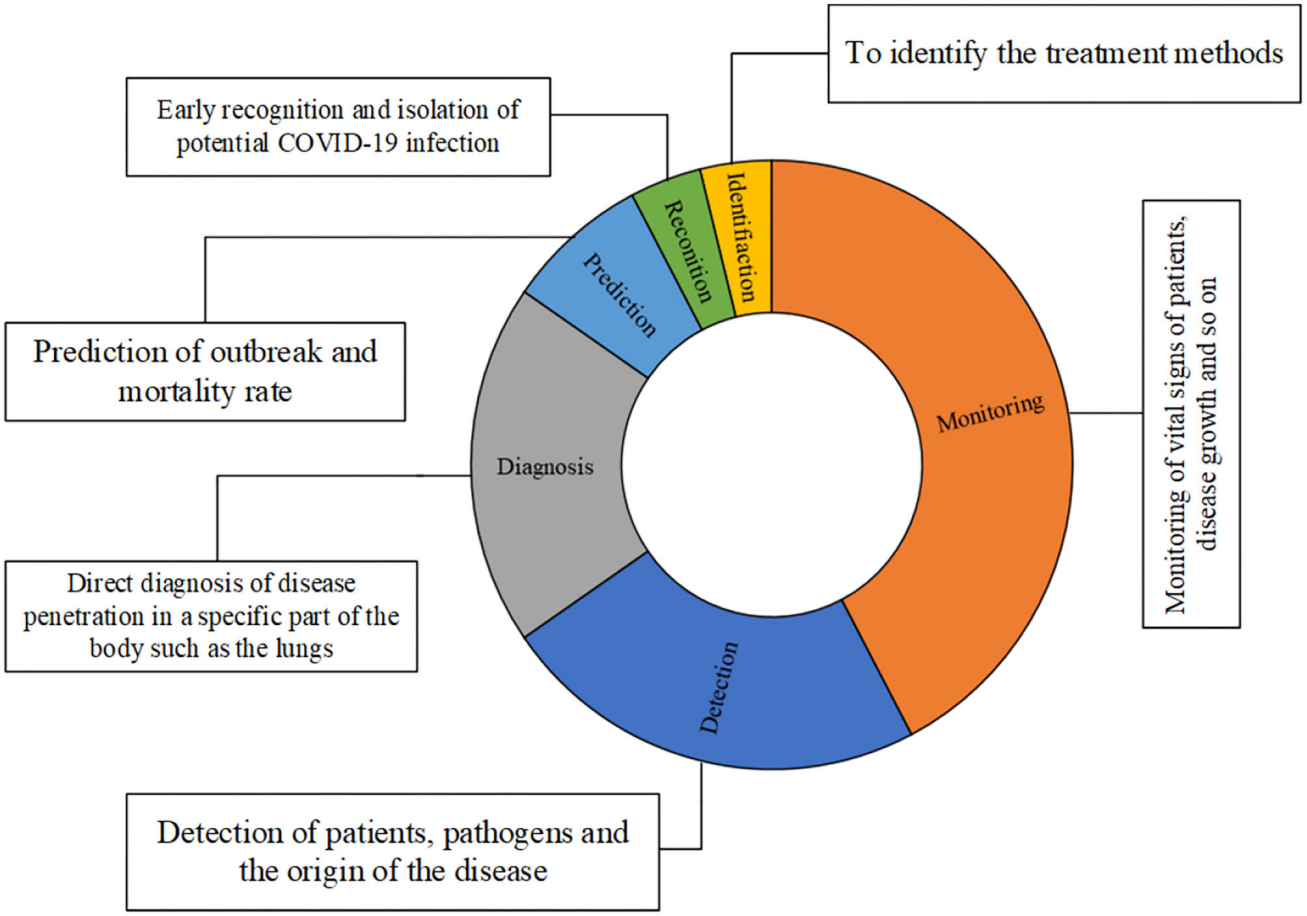
The share of each application type for IoT-based systems.

Figure 2 presents the contribution of different applications which are performed by IoT techniques to track COVID-19 related fields.

Figure 2 is generated based on Table 2 to present the main applications and their portions by studies for handling IoT in the COVID-19 pandemic. Monitoring, detection, and diagnosis are the main application of IoT-based techniques in tackling the COVID-19 pandemic. Monitoring can be performed in different ways. Accordingly, Roy et al. employed IoT as a real-time solution for monitoring COVID-19 outbreaks (66). Also, Otoom et al. employed IoT to provide monitoring and detection data using a real-time system to feed to the machine learning algorithms for further applications or handling (69). Singh et al. and Vedaei et al. used IoT as a tool for monitoring COVID-19 patients and their health condition in cooperating with an interconnected network (70, 71). Ashraf et al. proposed a smart edge surveillance system to monitor wearable smart gadgets which are operated according to IoT-based technology (72). Karmore et al. developed a Medical Diagnosis Humanoid to provide a complete diagnostic system for COVID-19 using IoT-based technology (74). De Vito et al. presented the outputs of a high-resolution A.Q. monitoring system based on an IoT-based technique (80).

Baskaran et al. used a non-contact infrared sensor to examine the body temperature to detect the patients with COVID-19 (73). Wang et al. exploited the social relationships in the platform of Social IoT to solve controlling issues of the COVID-19 epidemic by sharing the limited protective resources (77). Kumar et al. investigated an IoT based platform to prevent the spreading of COVID-19 (78). Kolhar et al. developed a platform of a decentralized IoT-based biometric based on a face detection platform for handling COVID-19 outbreaks (79). Aman et al. developed an architecture of IoT based framework for medical applications with respect to combat COVID-19 (38). Manalu et al. investigated the information technology to respond COVID-19 pandemic trend in accordance with the IoT technology (81).

**Figure 3:**
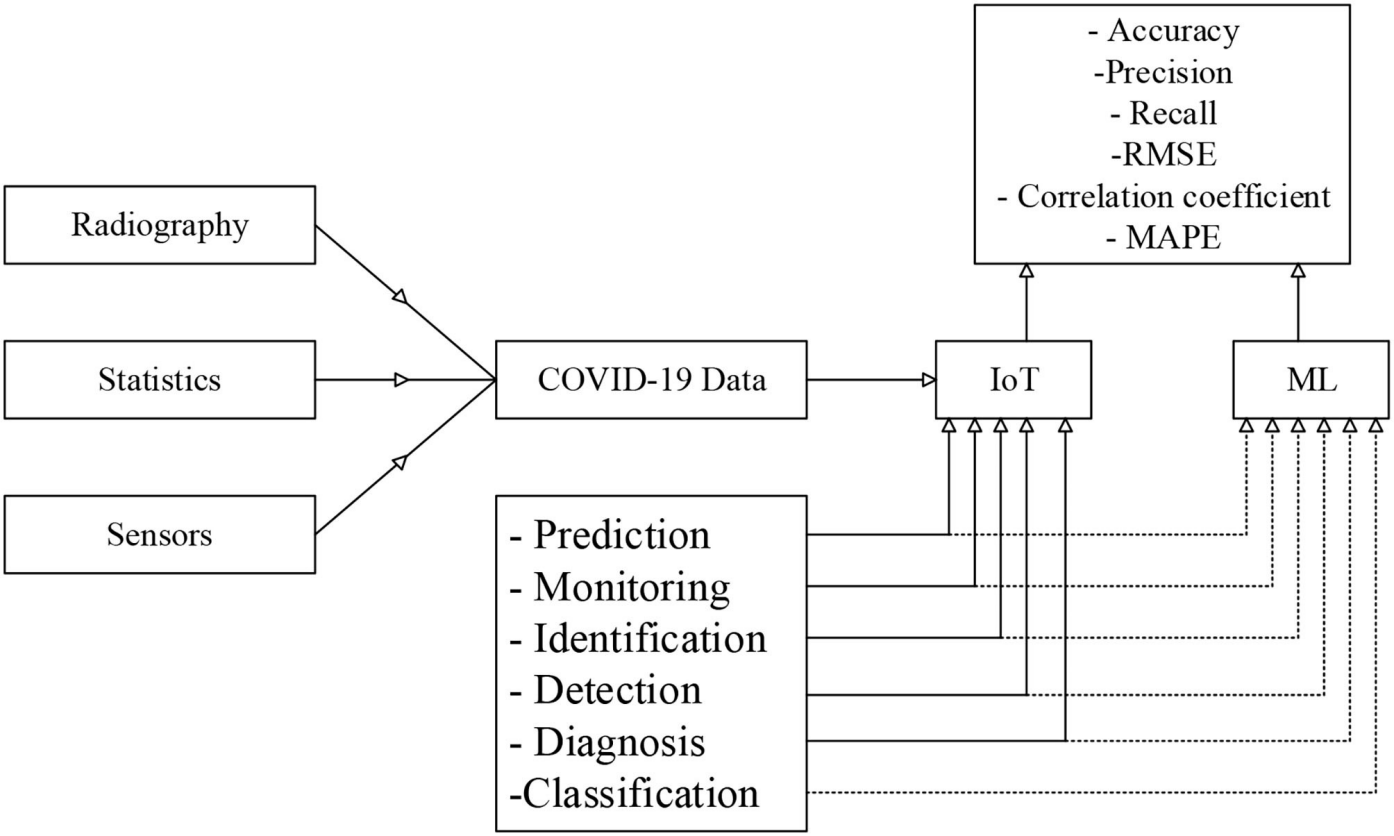
Main contributions of the current study.

Figure 3 presents the main contribution of these papers. According to the reviewed studies, the COVID-19 dataset can be imported from three main sources, including Radiography, statistics of health centers, and Sensors for prediction, monitoring, identification, detection, diagnosis, and classification purposes. The output of the techniques needs to be evaluated to confirm the approach performance and accuracy values. The frequently used parameters for performance analysis include Accuracy, Precision, Recall, R.M.S.E., Correlation coefficient and mean absolute percentage error. This can be considered a brief explanation of the main contribution of the present study. This study successfully presents the advantages and disadvantages of each technique for a specific task in handling the COVID-19 dataset and proposes the future perspectives. Also, this study can detect the main challenges and limitations.

There is a need to categorize the main applications of IoT and the relevance technique following COVID-19. Table 3 presents the study's main contributions to the application of IoT and integrated IoT-ML-based techniques. Based on Table 3, the exact application of each of the methods used can be extracted. It is also possible to find out which methodology is still available for which application can be considered a research opportunity for the future. Also, by carefully examining the different reasons for the tendency of each method to the fields shown in independent research, which can be considered necessary research and planning opportunities for policymakers in this field.

**Table 3:** The main contribution of the study for the application of IoT based techniques.

**Methodology**	**Prediction**	**Monitoring**	**Detection**	**Identification**	**Diagnostic**
IoT	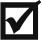	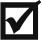	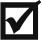		
IoT-DNN					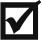
IoT-RCNN					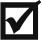
IoT-SVM		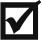		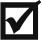	
IoT-ANN		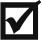		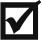	
IoT-Naïve Bayes		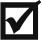		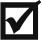	
IoT-K-NN		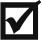		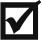	
IoT-DT		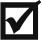		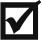	
IoT-Fog based		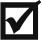			
IoT-Deep edge computing		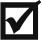			
Wireless sensors		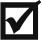			
IoT based S.E.I.R.			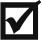		
IoT-IT		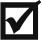	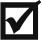		

**Figure 4:**
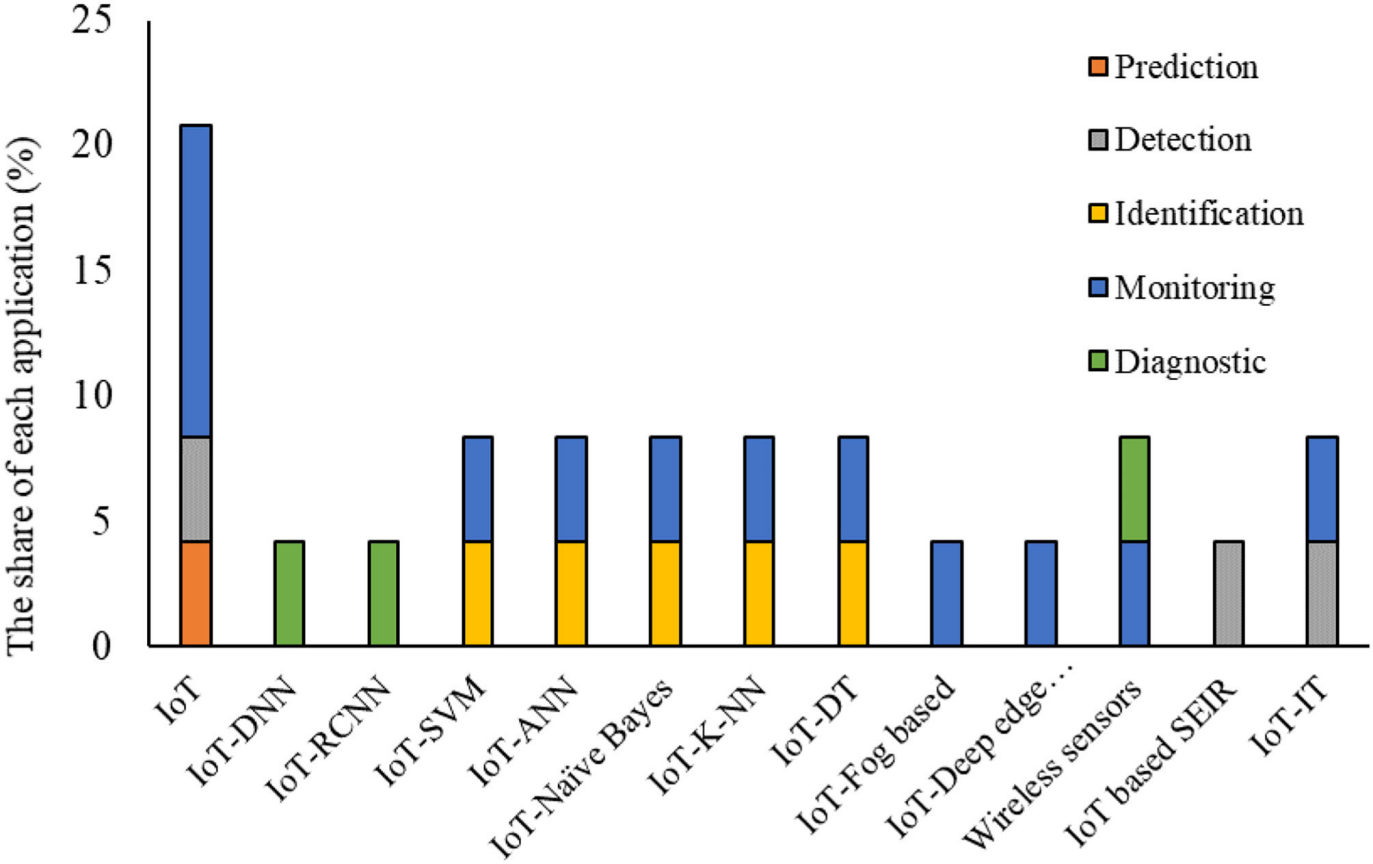
The share of each application (%).

As shown in Table 3, IoT-based technology requires ML-based techniques to complete the task. Figure 4 presents the share of each methodology in the applications by percentage.

As shown in Figure 4, IoT has been used more than other applications to monitor and detect COVID-19 cases. However, it has been less popular in the identification at the same time.

### ML Techniques for Pandemic Prediction of COVID-19

Utilizing the ML platform led to reducing the adverse effects of the disease and accelerating the healing process (62). The combination of A.I. and ML has led to advances in treatment, medication, screening, prognosis, contact tracking, and the drug/vaccine development process and reduced human intervention in medical performance (82). ML is also used as a tool for managing virtual queues to prevent crowds in physical waiting rooms or long queues. In addition, it is used to predict waiting times and implement calls in a privacy manner in conjunction with the cell phone platform (83).

The ML method is widely used in data analysis by intelligently producing an analytical model. This method is a subset of artificial intelligence that analyzes data and produces a model for estimating, categorizing, optimizing, predicting, identifying problems, and decision-making (84, 85).

New computing technologies have made the problems assessed by ML-based techniques today a little different from the way they are analyzed based on past technologies (86). These techniques began to evolve from pattern recognition to a comprehensive theory of the ability of computers to perform specific tasks without the need for special planning (87, 88).

**Figure 5:**
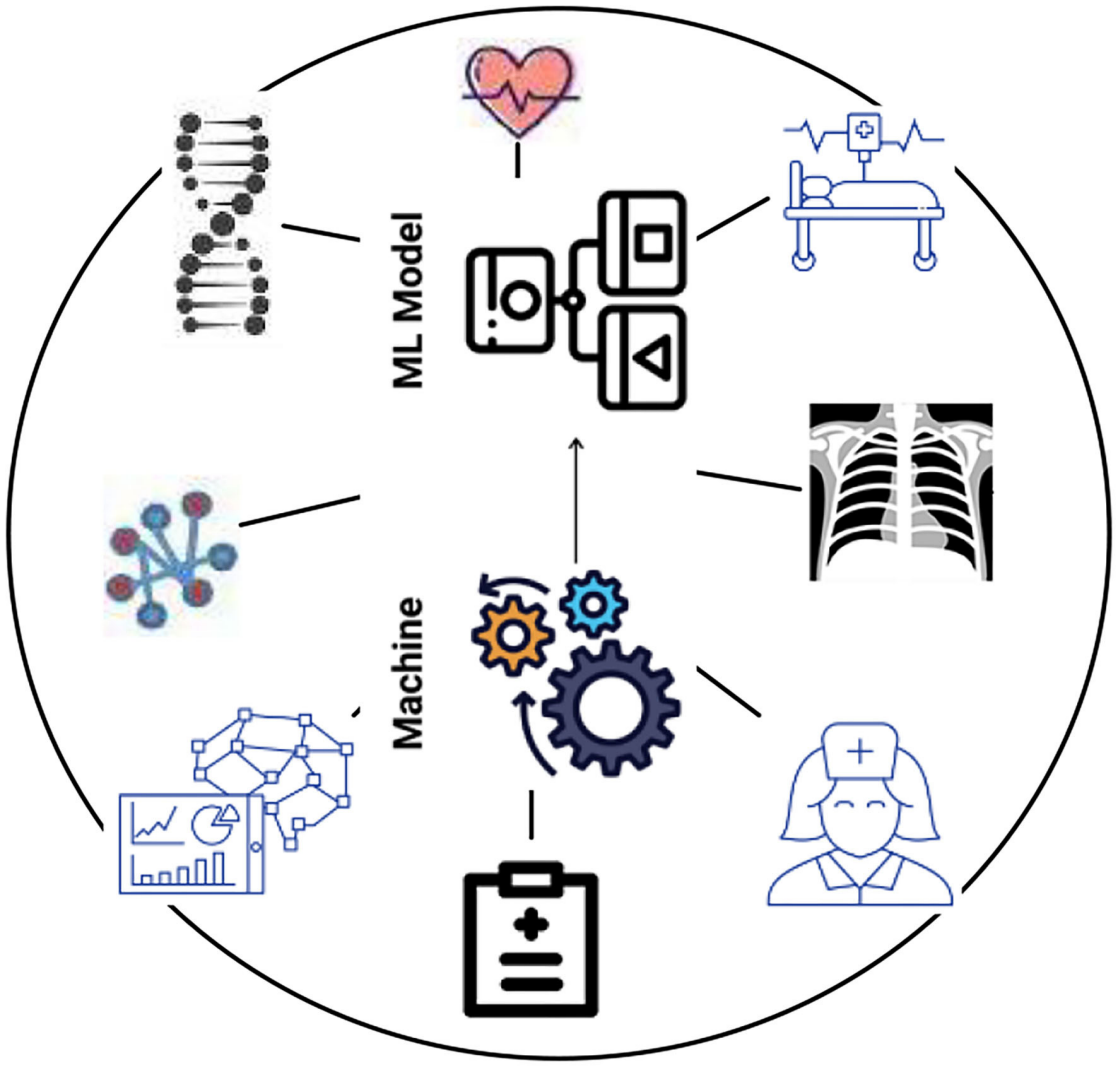
The main applications of ML-based techniques for medical science.

In the field of medicine and treatment, ML is known as one of the most practical tools for analyzing medical data, identifying, predicting, and even treating different situations. With the advancement of medical science in today's world and the production of large volumes of medical data, there is an urgent need to analyze this data (89). Figure 5 presents the main applications of ML-based techniques for medical science to tackle the COVID-19 pandemic. Identifying the prevalence, effective parameters in the eradication of the virus, identifying patients in the early stages, patients' pattern behaviors, and predicting outbreak and mortality rates can be considered practical and effective areas of ML-based techniques (90, 91).

Table 4 presents the highlighted studies for the application of ML-based techniques for handling COVID-19. Similar to Tables 3, 4 discuss them in four columns. The objective column briefly describes the main objective of each study. Methodology/proposed algorithm presents the main algorithm and procedure employed by each study. Keyword indicates the main points and concentration of the study and finally, application section presents the field of the application of each method.

**Table 4:** ML-based techniques for COVID-19.

**Order**	**Aim**	**Method**	**Dataset**	**Key words**	**Application**	**Reference**
1	To develop a mask face detection model	Deep transferring learning (ResNet50) as classifier and SVM to be compared with ensemble method	Image-based dataset	Deep transferring learning, SVM, and ensemble	Detection	(92)
2	To employ ML based platform as a healthcare application to proper decision making for COVID-19 detection	Integration of random forest, Gaussian nave bias and Generative adversarial network	Real-time processing of users' health data	Artificial intelligence, Cloud/fog computing, IoT	Detection	(93)
3	To propose an A.I. based technique integrated by C.T. scan and chest x-ray images to identify, and predict the positive infected patients	Pre-trained CNN	Chest X-ray and C.T. scan images	COVID-19, DT, X-ray images, AI	Identification and diagnosis	(94)
4	To employ a novel CNN architecture for classifying COVID-19 from chest X-rays.	CNN architecture	Chest X-ray	DL, CNN, mine data patterns	Classify and identification	(95)
5	To develop an AI based methods for fast diagnosis of COVID-19 cases	ResNet-101 in comparison with Radiology data	Chest X-ray radiography	AI, CNN, ResNet-101	Diagnosis	(96)
6	To detect COVID-19 promptly using CNN	CNN technique	Chest X-ray images	DL, CNN, Squeeze Net	Detection	(97)
7	To develop and test a new computer-aided diagnosis (CAD) to investigate COVID-19	CNN	Multi-center chest C.T. dataset	CNN, DL, CAD	Diagnosis	(40)
8	To propose an intelligence computer-aided model to support daily clinical applications	Convolution neural network (CNN) with SVM classifier architecture on chest X-ray	Chest X-ray	Medical decision support system; Deep learning	Detection	(98)
9	To develop an AI-based model for proper screening and monitoring of COVID-19	AD3D-MIL	Chest X-ray images	Screening, CAD, DL, ML	Monitoring	(99)
10	To present a CNN based technique for early COVID-19 diagnosis from chest X-ray	CNN	Chest X-ray	A.I., CNN, DL	Diagnosis	(39)
11	To investigate a medical decision support system by CNN	CNN	Chest X-ray images	Decision support; CNN; DL; ML	Diagnosis	(100)
12	To propose an intelligent methodology to diagnosis the COVID-19 cases	The multi-criteria decision-making (M.C.D.M.) using T.O.P.S.I.S. in the presence of SVM based classifier	Chest X-ray Dataset	COVID-19 diagnostic, machine learning, benchmarking; TOPSIS,	Diagnosis	(101)
13	To study the utility of A.I. in a prompt and accurate diagnosis of COVID-19 in the presence of chest X-ray images	Pre-trained CNN	Chest X-ray images	AI; COVID-19; machine learning, Convolutional Neural Networks	Diagnosis	(102)
14	ML-based classification approach for handling COVID-19	Extreme gradient boosting (XGBoost) model	Eight pathogenic species	Dinucleotide frequencies, feature representations, genomic signatures, human pathogens, ML, extreme gradient boosting	Classification	(103)
15	ML-based classification algorithm for handling infectious diseases, such as COVID-19	KNN, SVM, D.T. and L.R.	Wi-Fi signals data	Machine learning, classification, COVID-19,	Classification	(104)
16	To detect the COVID-19 cases using RNN technique	L.S.T.M. architecture of R.N.N. method for detection based on Cough sound, Breathing sound and voices	Speech and sound analysis dataset	AI, DL, RNN	Detection	(105)
17	To present a fuzzy rule basing system to predict COVID-19 daily cases	Fuzzy rule based	Daily cases data from the Turkish republic health ministry	COVID-19, A.I., fuzzy rule based inference	Prediction	(106)
18	To present a multi-scale discriminative segmentation network to detect COVID-19	MSD-Net	COVID-19 CT segmentation dataset	COVID-19, CT, DL	Diagnosis	(107)
19	To develop a hybrid A.I. technique for the prediction of COVID-19	Integrated natural language processing module and the L.S.T.M.	The epidemic data of several typical provinces and cities in China	COVID-19, prediction, epidemic model, hybrid A.I.,	Prediction, detection	(108)
20	To present a solution for identifying pneumonia using C.X.R. images	GCNN	CXR images	G.C.N.N., Computed Tomography, Chest X-Ray, A.I.	Classification	(109)
21	To examine the emotions expressed by people using social media to track and diagnosis sentiment behind COVID-19	LR, Multinomial Naïve Bayes, DT, RF, SVM and XGBoost classifiers	Fetch data from social media platform	Twitter; emotions; sentiment analysis; pandemic; domain-specific; COVID-19; ML; dataset	Detection	(110)
22	To propose an ML-based approach for the forecasting of COVID-19 cases	M.L.P. and A.N.F.I.S.	Outbreak dataset from WHO	ML, COVID-19 cases, prediction, detection	Detection	(91)
23	To develop hybrid ML-based technique for the globally prediction of COVID-19 cases	Multilayered perceptron integrated by gray wolf optimizer	Outbreak dataset from WHO	Machine learning, COVID-19 cases, prediction, detection	Detection	(90)

**Figure 6:**
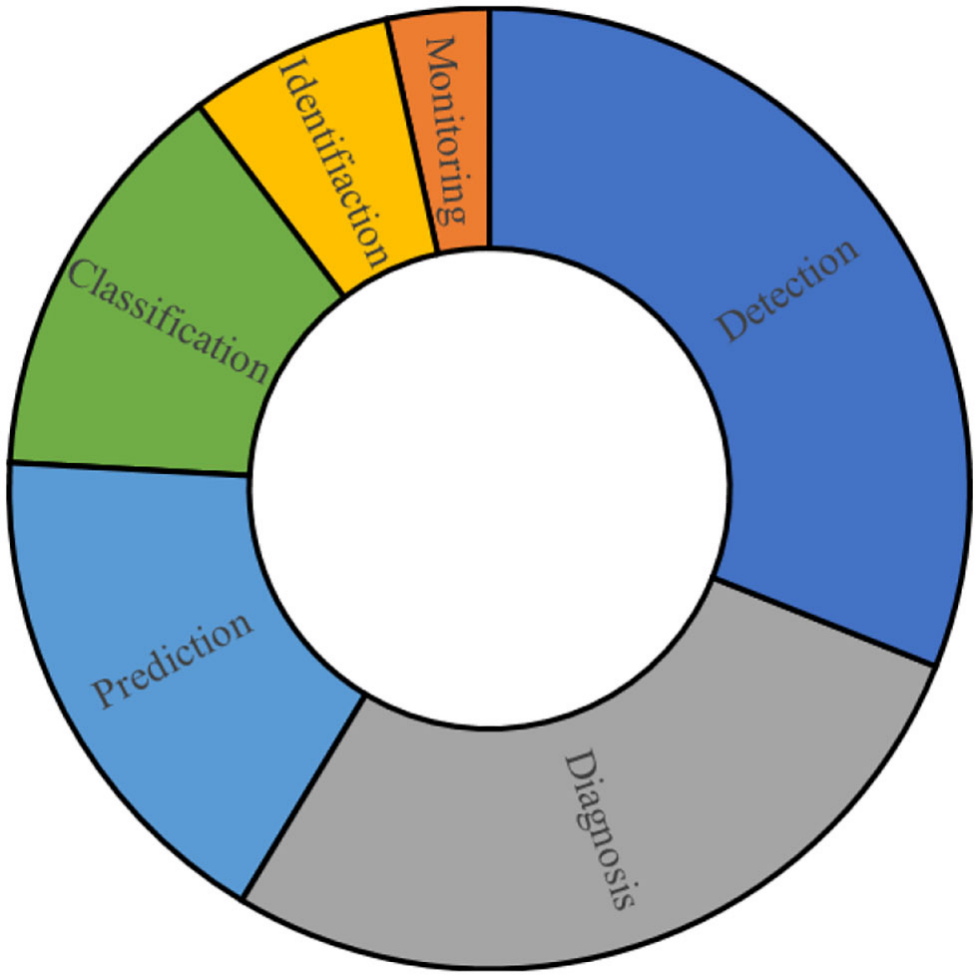
The share of each application type for ML-based systems.

According to Table 4, ML-based techniques are employed for detection, identification, monitoring, diagnosis, prediction, and classification purposes in the presence of the COVID-19 dataset. Figure 6 presents the summary of each application separately. Singh and Kaur employed an ML-based platform using hybrid random forest, Gaussian Naïve Bayes, and Generative adversarial network as a healthcare application to detect COVID-19 cases (93). Vinod et al. developed a pre-trained CNN method as an ML-based technique integrated using C.T. scan and chest x-ray images to identify, detect, and predict the positive infected patients (94). Ardakani et al. developed an ML-based technique (ResNet) for fast diagnosis of COVID-19 cases compared to radiology data (96). Polsinelli et al. developed a study to detect COVID-19 promptly using CNN as a frequently used DL-based architecture (97). Nour et al. proposed an intelligence computer-aided model based on CNN with SVM classifier architecture on chest X-rays to support daily clinical applications (98). Chowdhury et al. investigated the utility of A.I. in the rapid and accurate detection of COVID-19 in the presence of chest X-ray images (102). Sethi et al. employed Logistic Regression (L.R.), Multinomial Naïve Bayes, Decision Tree (D.T.), Random Forest (R.F.), SVM, and XGBoost classifiers to analyze the emotions expressed by people using social media to monitor and detect sentiment behind COVID-19 (110). Ardabili et al. developed ML-based techniques for the prediction of COVID-19 outbreaks (91). In another study, Ardabili et al. also employed a hybrid ML-based technique (Multilayered perceptron integrated by gray wolf optimizer) for the global prediction of COVID-19 cases (90). In addition, Loey et al. employed the DL-based ResNet method in the presence of an SVM-based classifier to detect a masked face (92).

According to Figure 6, detection, diagnosis, and prediction can be considered as the main categories of the application of ML-based methods in COVID-19. In general, one of the main sections of analyzing IoT-based and ML-Based techniques applied for a specific field is their evaluation in terms of accuracy, error, or in other word performance of the model. Table 5 presents the evaluation criteria employed for each model.

**Table 5:** The main evaluation criteria for analyzing the performance of models.

	**Accuracy**	**Recall**	**Precision**	**AUC**	**Sensitivity**	**specificity**	**Determination coefficient**	**RMSE**	**MAPE**	**MAE**	**F1-score**
Deep transferring learning (ResNet50)	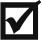										
RF-NB-GAN		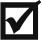	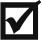								
CNN	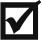	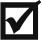	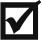	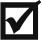					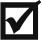		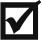
ResNet-101	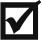				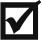	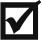					
AD3D-MIL	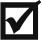			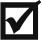							
T.O.P.S.I.S.							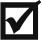				
XGBoost	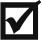										
kNN	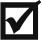										
SVM	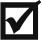										
D.T.	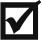										
L.R.	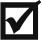										
L.S.T.M.	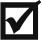			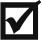							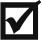
Fuzzy							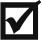	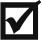		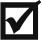	
MSDN					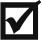	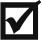					
Naïve Bayes	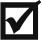		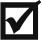		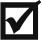						
R.F.	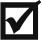										
MLP											
ANFIS							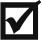	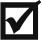			
MLP-GWO							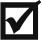		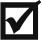		
IoT (Medical based)	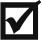			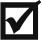							
Fog-based	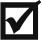										
Deep edge computing	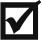										
Wireless sensors	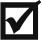										
IoT based S.E.I.R.	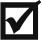										
I.T.	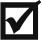										

According to Table 5, accuracy, followed by the recall and precision parameters has owned the highest portion of the evaluation criteria employed for analyzing COVID-19 based dataset using IoT and ML-based techniques. In the following, Table 6 is generated from Table 4 for indicating the share of each ML-based technique for each application and their main contributions. According to Table 6, ResNet as an architecture of deep learning methods followed by CNN, XGBoost, SVM, D.T., and L.R. has been used more often to tackle work with COVID-19 related data.

**Table 6:** The main contribution of ML-based techniques in COVID-19 applications.

	**Prediction**	**Monitoring**	**Diagnosis**	**Identification**	**Detection**	**Classification**
ResNet50	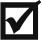	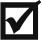	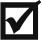	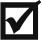	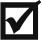	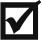
RF-Naïve bayes-GDN					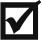	
CNN			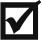	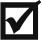	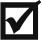	
ResNet-101			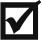			
AD3D-MIL		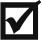				
T.O.P.S.I.S.			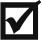			
XGBoost					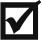	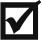
kNN						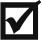
SVM					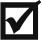	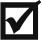
D.T.					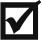	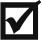
L.R.					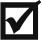	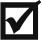
L.S.T.M.	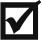				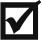	
Fuzzy	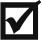					
Multi-scale discriminative network			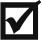			
G.C.N.N.						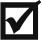
Naïve Bayes				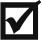		
R.F.					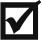	
M.L.P.	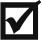					
A.N.F.I.S.	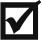					
MLP-GWO	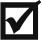					

**Figure 7:**
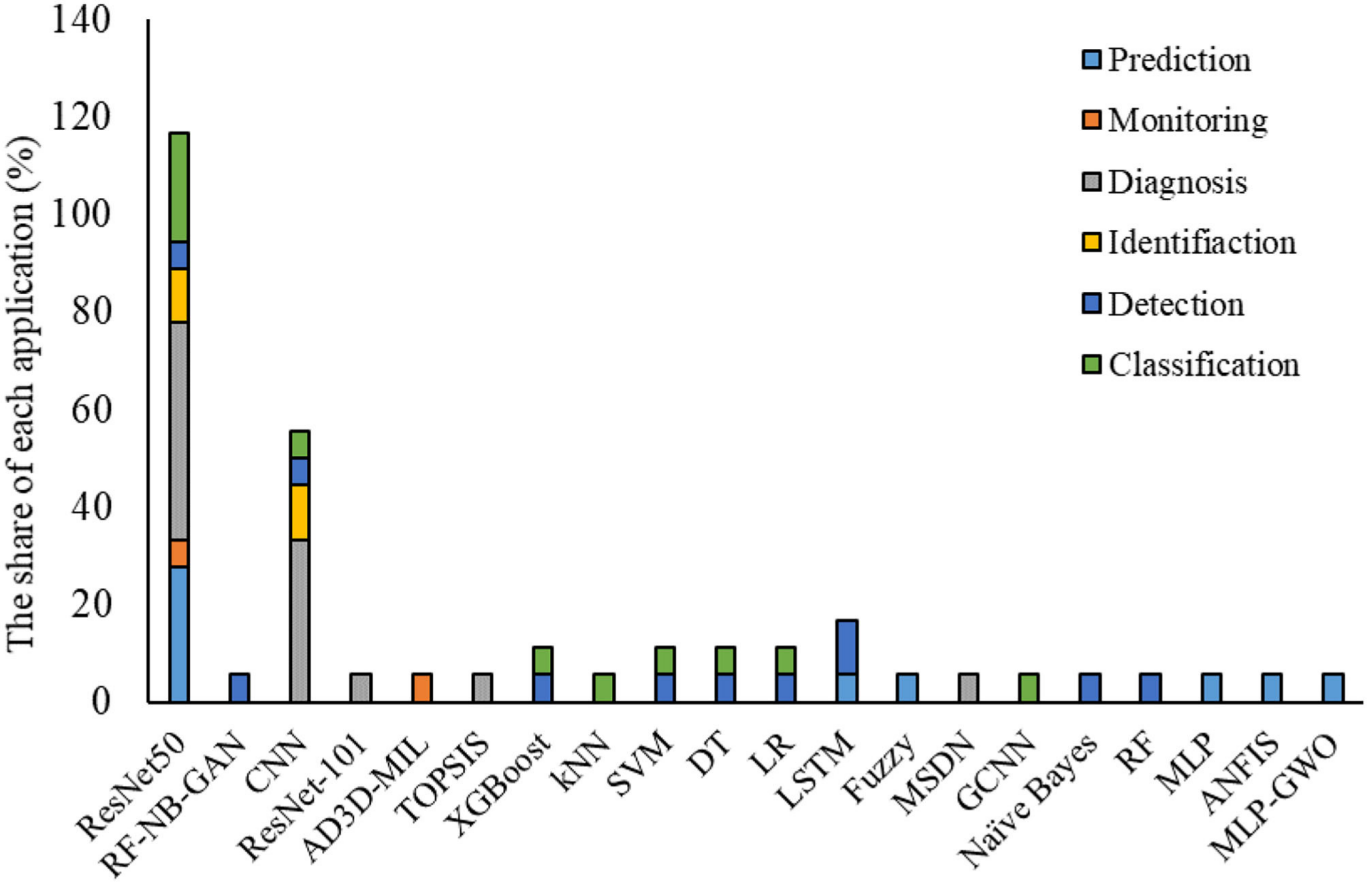
The share of each application (%).

Figure 7 presents the share of different ML methods for different tasks to tackle the COVID-19 pandemic. As is clearly indicated in this figure, ResNet, followed by CNN, is the most common application of ML in this field. This can be due to the model's nature for handling different applications like monitoring, detection, identification, classification, and diagnosis. In comparison, other methods can do a limited number of applications.

### Evaluation Criteria

Models developed using ML and IoT-ML require an evaluation step for recognizing their performance and accuracy values. According to the studies reviewed, the most effective and frequently used evaluation criteria are including Accuracy, Recall, Precision, Root mean square error (R.M.S.E.), Correlation coefficient and Mean absolute percentage error (M.A.P.E.). These criteria compare the models' output and actual values and provide a comparison score (90, 91). In the present study, we employed the criteria values reported by each study for evaluating and comparing the models. Table 7 presents the main criteria for evaluation.

**Table 7:** The main evaluation metrics.

$Accuracy\;=\;\frac{True_{p}+True_{n}}{True_{p}+True_{n}+False_{p}+False_{n}}$	Where *True*_*p*_ denotes the true positives, *True*_*n*_ the true negatives, *False*_*p*_ the false positive, and *False*_*n*_ the false negatives.	(1)
$Recall\;=\;\frac{True_{p}}{True_{p}+False_{n}}$	Where *True*_*p*_ denotes the true positives and *False*_*n*_ the false negatives.	(2)
$Precision\;=\;\frac{True_{p}}{True_{p}+False_{p}}$	Where *True*_*p*_ denotes the true positives and *False*_*p*_ the false positives.	(3)
$RMSE\;=\;\sqrt{\frac{1}{N}\sum_{i\;=\;1}^{N}(x_{i}-\hat{x_{i}}{)}^{2}}$	Where *N* denotes the total number of samples, *x*_*i*_ the actual samples, and ${\hat{x}}_{i}$ the predicted samples.	(4)
$Correlation\;Coefficient\;=\;\frac{Cov(x,\;\hat{x})}{\sigma_{x}\sigma_{\hat{x}}}$	Where *x* refers to actual samples, $\hat{x}$ to predicted samples, $Cov(x,\;\hat{x})$ to the covariance between *x* and $\hat{x}$, and σ to the standard deviation (calculated for both *x* and $\hat{x}$)	(5)
$MAPE=\frac{100}{N}\sum_{i=1}^{N}\left|\frac{x_{i}-{\bar{x}}_{i}}{x_{i}}\right|$	Where *N* denotes the total number of samples, *x*_*i*_ the actual samples, and ${\hat{x}}_{i}$ the predicted samples.	(6)

### Main Findings and Evaluations

This section presents the main findings of IoT based techniques (Table 8) and ML-based techniques (Table 9). Each table includes two main columns called findings and pros. and cons.

**Table 8:** The main findings of the study for the application of IoT-based techniques.

**Order**	**Findings**	**Pros. and Cons**.	**Reference**
1	The proposed solution can identify and track the infected individual and successfully tracks all people who are in the area of disease spread	This framework integrates symptom information as a rapid and efficient approach, thus tracking the prevalence of the disease	(66)
2	DL applications are vulnerable to coronavirus attacks	The method is very vulnerable and requires further studies	(67)
3	The model provides an accuracy of 98% for detection	Combining DL and the IoT makes it easier for radiologists to control the spread of the virus	(68)
4	According to results, all the techniques, except the Decision Stump, OneR, and ZeroR provided accuracies values more than 90%	The proposed platform reduced the communicable diseases using early detection of cases and provided tracking the recovered cases, and a better understanding of the infections	(69)
5	IoT reduces clinical cost and optimizes treatment outcome of the patients	The platform improves patient satisfaction and decreases readmission rate in the hospital	(70)
6	The system can assist tracking the daily activities and decrease the risk of exposure to the COVID-19	The app announces the user to keep a physical distance of 2 m. Also, a Fuzzy-based technique evaluates the environmental risk and user health to estimate the risk of real time spreading. This platform can successfully reduce the coronavirus spread	(71)
7	The platform detects and tracks the infected person	The platform tracks COVID-19 and improves infected person and keeps the dataset for further analysis	(72)
8	The provided package enhances the testing process for increasing the efficiency of the system	This approach will increase the maximum collaboration from the employees	(73)
9	This platform is a cost-effective, safety-critical mobile robotic technology and successfully copes with diagnosis task Also the multiple diagnostic devices increases the detection accuracies	The system effectively provides a complete diagnosis and figuring out COVID-19 patients also contains multiple diagnostic devices, without any need for human interferences	(74)
10	The robot technology protect virus affected persons. The system is also recognizing the patient's Gesture and tracking the instructions	The robot collects data from patient performs tasks without image processing system	(75)
11	IoT-based technology prevent the global pandemic	Improves the control and tracking of a fast-spreading virus such as coronavirus	(76)
12	The proposed methodology is sustainable for disease tracking by an early identification of cases	This technique can successfully handles both governments and other decision-making authorities	(77)
13	This system improves the decision-making procedure	The system is connected through cloud computing and effectively supports the real-time data	(78)
14	Edge computing improved the findings on the decentralized load of face recognition	The platform enhances the robustness of detection and diagnosis	(79)
15	The proposed system could successfully cope with the task	IoT equipped ML can successfully save, and visualize monitoring the volunteers	(80)
16	This study suggests that integrated and hybrid techniques will follow up the near future, using simulation, and forecasting purposes	A higher degree of safety and privacy for humanity	(38)
17	The platform employed for the study have an effective role in the success of pandemic handling	The platform increases accessibility to the proper dataset	(81)

**Table 9:** The main findings of the study for the application of ML-based techniques.

**Order**	**Results**	**Pros. and cons**.	**Reference**
1	The SVM classifier in the presence of R.M.F.D., S.M.F.D. and L.F.W. dataset achieved 99.64, 99.49 and 100% testing accuracy values.	The proposed model provided lowest processing time and highest accuracy	(92)
2	Recall = 0.93, Precision = 0.871 with lower processing time	The system is cost-effective by reducing processing time and sustainable by increasing the accuracy values considerably. The proposed framework can also be used to prioritize patients who require an ambulance.	(93)
3	Accuracy = 93% and recall score = 88% using chest x-ray images	The proposed method can successfully help radiologist's prompt detection of coronavirus cases	(94)
4	Accuracy (97.94 %) and AUC (98.39 %)	A channel-shuffled dual-branched CNN architecture can effectively learn salient features and increases the accuracy and precision values of the modeling	(95)
5	Sensitivity = 100%, specificity = 99.02% and accuracy = 99.51% and for radiology data, sensitivity 89.21%, specificity = 83.33% and accuracy = 86.27%	This model is low cost and is used as a complementary method during C.T. imaging	(96)
6	Accuracy = 85.03%, sensitivity = 87.55%, specificity = 81.95%, precision = 85.01% and F1-core = 86.20%	Higher classification rate by analyzing thousands of images	(97)
7	Accuracy = 94.5%, confidence interval = 95%, sensitivity = 98.4% and specificity = 98.0%	Develops a DL-based CAD scheme of chest X-ray images and improves detecting COVID-19 infected	(40)
8	Accuracy = 98.97%, sensitivity = 89.39%, specificity = 99.75%, and an F-score = 96.72%	Reduces the misdiagnosis rates, and improves evaluation rates and detects positive COVID-19 infections	(98)
9	Accuracy = 97.9%, AUC = 99.0%, and Cohen kappa score = 95.7%.	Reliable screening of COVID-19 from chest CT	(99)
10	96% of accuracy	The proposed model performance is clinically validated with expert radiologists	(39)
11	Accuracy of 99.62 and 96.70%. Average recall value of 99.63 and 96.69%, respectively, for binary and multiclass	Automated medical diagnostics for enhancing decision making rates	(100)
12	Correlation coefficient = 0.9899	providing significant variance for each criterion	(101)
13	Accuracy = 99.7%, precision = 99.7%, and sensitivity = 99.7%	Improving the speed and accuracy of COVID-19 detection	(102)
14	86% accuracy for the task of classifying	The proposed model could successfully improve the classification accuracy	(103)
15	Accuracy of 88, 91, 87 and 89% for kNN, SVM, D.T. and L.R., respectively	The proposed method can be applied anywhere, without prior training or calibration	(104)
16	F1-score of 97.9, 98.8, and 92.5%, A.U.C. of 97.4, 98.8, and 84.4% and accuracy of 97, 98.2, and 88.2%, respectively, for Cough sound, Breathing sound and voices, respectively.	To improve the COVID-19 detection through a cost-effective approach	(105)
17	R^2^ = 0.96, RMSE = 254, MAE = 186	The proposed method could successfully estimate the number of daily cases	(106)
18	Sensitivity and specificity of 0.8645, and 0.9889.	This model provides automated and accurate segmentation of C.T. images	(107)
19	MAPEs = 0.52, 0.38, 0.05, and 0.86%, respectively for the next 6 days in Wuhan, Beijing, Shanghai, and countrywide	To minimize the errors of the prediction and to enhance the detection efficiency	(108)
20	Accuracy = 98.84%, Precision = 93%, Sensitivity = 100%, and Specificity = 97.0%	The proposed model improved classification rate in comparison with ReseNet18, ReseNet50, Squeeze net, DenseNet-121, and Visual Geometry Group	(109)
21	Accuracy for both SVM and Decision Tree could provide the maximum value by average value of 93%	Higher accuracy for perceiving the perception of people infected by COVID-19	(110)
22	R.M.S.E. and CC values for five countries including, China, Italy, U.S.A., Iran and Germany	The proposed models enhanced the forecasting rate of COVID-19 cases	(91)
23	MAPE = 13.15% and CC = 0.99	The proposed models increased the forecasting rate of COVID-19 cases	(90)

According to Table 8, most of these studies lack numerical analysis for the method's performance. One of the main reasons can be the nature of the IoT technique, which goes through a practical process and shows its performance in practical applications and does not need to provide numerical statistics. In all these applications, IoT could successfully cope with the task. IoT provided a fast and efficient approach to tracking the disease spread (66). On the other hand, it can be employed as a real-time framework to minimize the impact of communicable diseases through the early detection of cases (67). In the study by Singh et al. IoT technology successfully increased patient satisfaction and reduced the readmission rate in the hospital (70). However, there is a need to integrate IoT platforms with ML-based techniques for detection purposes. In the study by Rahman et al. DL applications with IoT platforms provided promising findings to detect A.E. attacks. However, there is a need for further research, attention, and implementation of appropriate defense mechanisms, safeguards, and controls (67). Kolhar et al. employed Multi-task Cascaded Convolutional Network architecture (M.C.C.N.N.), and findings claimed that the efficiently integrated by Raspberry Pi increased the robustness of detection and recognition (79).

**Figure 8:**
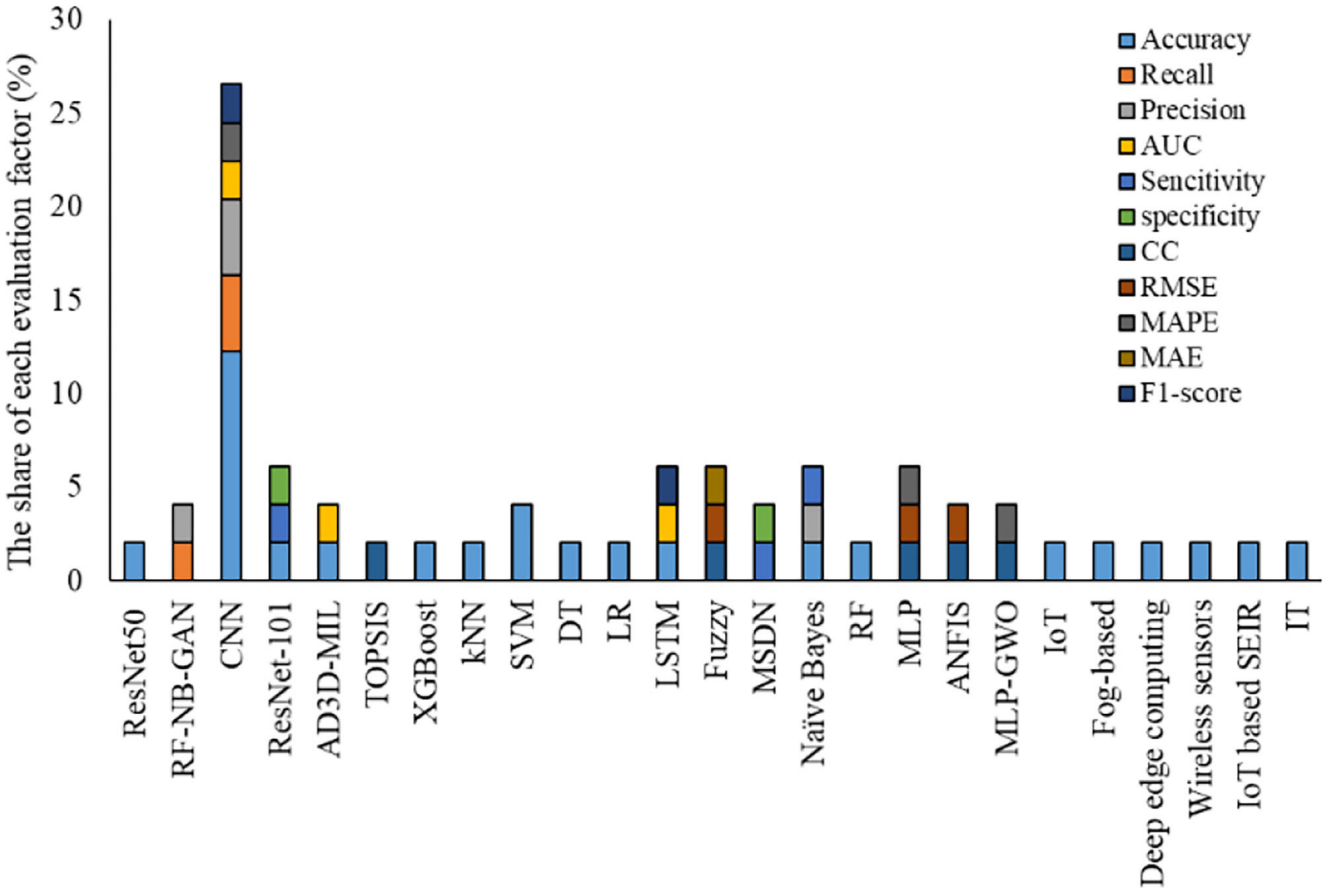
The share of each evaluation factor (%) for analyzing results.

**Figure 9:**
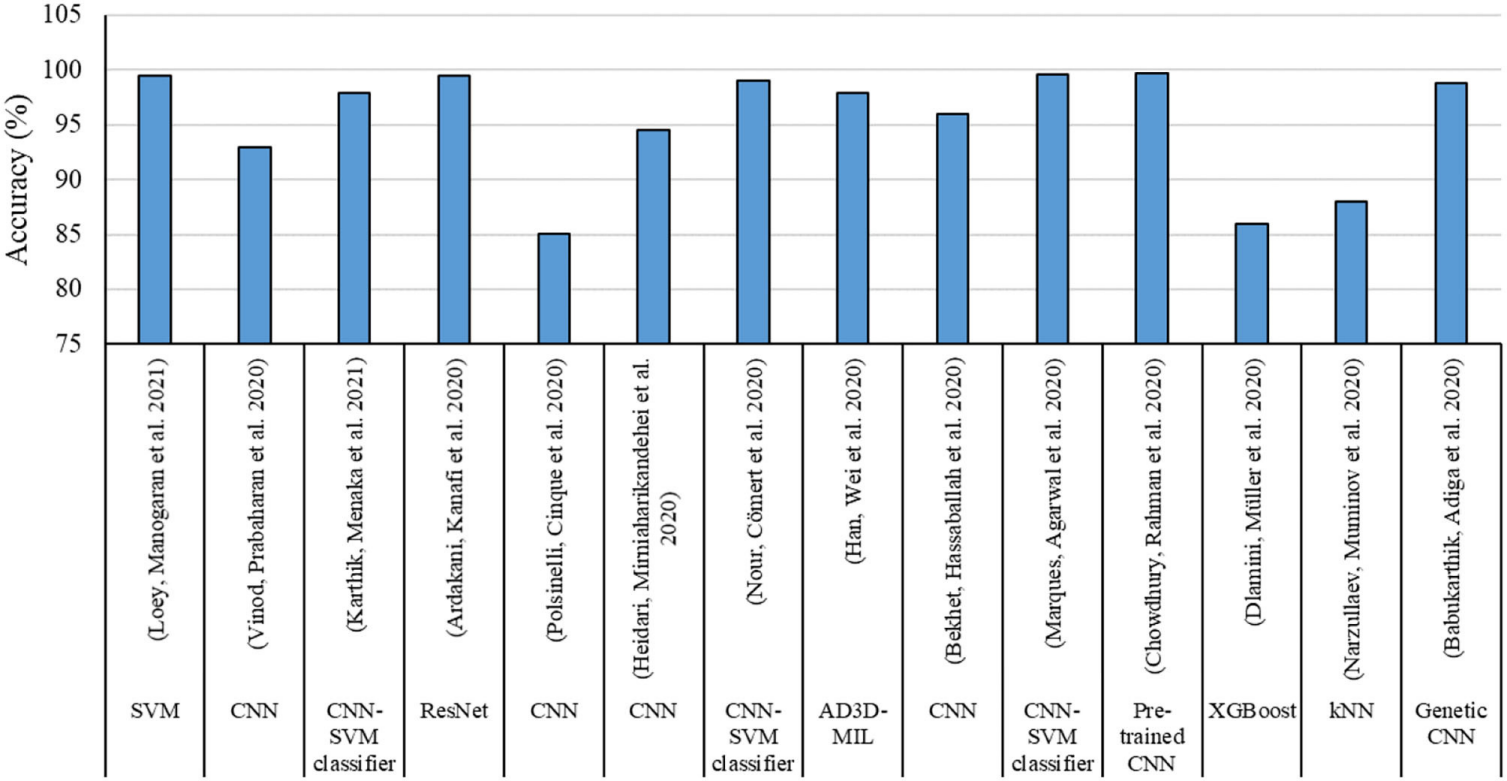
Accuracy values.

According to the findings given in Table 9, the most share of studies developed by ML-based techniques for handling COVID-19 based datasets provided performance criteria. The most share of the performance criteria, according to Figure 8 is related to the accuracy factor. Accuracy factor is a general and normalized factor. Therefore, it can be employed for comparing the ML-based methods with different datasets. Figure 9 presents the accuracy values for each model for comparing their performance in handling the COVID-19 dataset.

Figure 9 indicates CNN with SVM classifier, Genetic CNN, and pre-trained CNN followed by ResNet, provided highest accuracy values. On the other hand, the lowest accuracy was related to single CNN followed by XGboost and K.N.N. techniques.

### Challenges and Limitations

Nowadays, when the world is struggling with COVID-19 disease, every innovation and technology is used to fight this disease. Like many other areas, healthcare requires the support of new technologies such as IoT, and ML. Exploring the disease-related dataset, data preparation, prevention, and control of infectious diseases has become one of the main purposes of A.I. IoT and ML have a vital personality in better understanding, dealing with the COVID-19 crisis, and discovering the COVID-19 vaccine. ML-based technology allows computers to predict the pattern and speed of disease transmission with their intelligence and by mimicking large amounts of data. A.I. uses information from people with coronary heart disease, and improved and dead people as tracking data.

To combat the spread of the corona virus, IoT-based methods of communicating with patients provide transparency and a better understanding of how the virus is spread and strengthen the treatment and research process. ML is one of the new technologies for tracking the spread of the virus and finding effective parameters in it. The ML method can successfully identify high-risk patients and predict the necessary measures to deal with possible infections to reduce the point of the effect of the disease. In addition, ML-based methods can estimate the risk of patient mortality through previous analysis. This technique improves patients' planning, treatment, and reduction and is a complementary medical tool that works with data and evidence. On the other hand, this technology improves decision-making and reduces the cost of treatment and diagnosis. At the same time, in medical imaging, ML tools help to recognize the patterns in the images and strengthen the ability of radiologists to diagnose the possibility of disease and early diagnosis of the disease.

One of the main limitations of IoT, and ML-based techniques for applications in COVID-19 is the lack of a complete dataset. This can be due to the unique development of models by limited data for a specific application within the same data field. The purpose of using IoT, A.I., or ML-based techniques is to solve a specific problem in the real world with a real application that requires the use of special hardware and equipment. There are limitations in the cost and availability of developing and equipping communication hardware in therapeutic, diagnostic, estimation, and forecasting applications for IoT technology or ML-based techniques. Also, there are limited best practices available for IoT developers. The lack of IoT edge authentication and licensing standards has led to restrictions on the application and enactment of laws, regulations, and policies in the use of this technology, and this has led to the absence of IoT-based incident response activities as the best methods. All of these limitations mean that there is still no focus on identifying ways to gain situational awareness of the security of IoT assets in a medical complex.

## Discussion

According to the reviewed studies, the COVID-19 dataset can be imported from three primary sources: radiography, health centers' statistics, and Sensors for prediction, monitoring, identification, detection, diagnosis, and classification purposes. The output of the techniques needs to be evaluated to confirm the approach performance and accuracy values. The frequently used parameters for performance analysis include Accuracy, Precision, Recall, R.M.S.E., Correlation coefficient, and mean absolute percentage error. This can be considered a brief explanation as the main contribution of the present study. This study successfully presents the advantages and disadvantages of each technique for a specific task in handling the COVID-19 dataset and proposes future perspectives. Also, this study can detect the main challenges and limitations.

It is also possible to find out which methodology is still available for which application can be considered a research opportunity for the future. Also, by carefully examining the different reasons for the tendency of each method to the fields shown in independent research, which can be considered necessary research and planning opportunities for policymakers in this field.

The presence of the ML platform led to reducing the adverse effects of the disease and accelerating the healing process, advances in treatment, medication, screening, prognosis, contact tracking, and the drug/vaccine development process, and reduced human intervention in medical performance as a tool for the management of virtual queues to prevent crowds in physical waiting rooms or long queues. It is used to predict waiting times and implement calls privately with the cell phone platform.

Based on the studies conducted in this study, we achieved the following results:

- IoT has been used more than other applications to monitor and detect COVID-19 cases. In contrast, it has been less popular in the identification.
- ML method is widely used in data analysis by producing an analytical model intelligently for estimating, categorizing, optimizing, predicting, identifying problems, and decision making.
- New computing technologies have made the problems assessed by ML-based techniques, began to evolve from pattern recognition to a comprehensive theory of the ability of computers to perform specific tasks without the need for special planning.
- Identifying the prevalence, effective parameters in eradicating the virus, identifying patients in the early stages, patients' pattern behaviors, and predicting outbreak and mortality rates can be considered practical and compelling areas of ML-based techniques.
- Detection, diagnosis, and prediction can be considered the main categories of the application of ML-based methods in COVID-19. In general, one of the main sections of analyzing IoT-based and ML-Based techniques applied for a specific field is their evaluation in terms of accuracy, error, or performance of the model.
- Accuracy, followed by the recall and precision parameters, has the highest portion of the evaluation criteria employed for analyzing the COVID-19 dataset using IoT and ML-based techniques. ResNet, as an architecture of deep learning methods followed by CNN, XGBoost, SVM, D.T., and L.R., has been used more often to tackle work with COVID-19 related data.
- Resnet follows CNN is The most common use of ML to contribute various methods for different tasks to combat Pandemic COVID-19. This trend can be due to the model's nature for handling different applications like monitoring, detection, identification, classification, and diagnosis. At the same time, other methods can do a limited number of applications.
- Models developed using ML and IoT-ML require an evaluation step for recognizing their performance and accuracy values. According to the studies reviewed, the most effective and frequently used evaluation criteria include Accuracy, Recall, Precision, Root mean square error (R.M.S.E.), Correlation coefficient, and Mean absolute percentage error (M.A.P.E.). These criteria compare the models' output and actual values and provide a comparison score (90, 91). In the present study, we employed the criteria values reported by each study for evaluating and comparing the models.
- Most of these studies lack numerical analysis for the method's performance. One of the main reasons can be the nature of the IoT technique, which goes through a practical process and shows its performance in practical applications and does not need to provide numerical statistics. In all these applications, IoT could successfully cope with the task. IoT provided a fast and efficient approach to tracking the disease spread. On the other hand, it can be employed as a real-time framework to minimize the impact of communicable diseases through the early detection of cases.
- The most share of studies developed by ML-based techniques for handling COVID-19 based dataset provided performance criteria. The most share of the performance criteria is related to the accuracy factor. The accuracy factor is general and normalized. Therefore, it can be employed for comparing the ML-based methods with different datasets.
- CNN, SVM classifier, Genetic CNN, and pre-trained CNN followed by ResNet provided the highest accuracy values. On the other hand, the lowest accuracy was related to single CNN, followed by XGboost and K.N.N. techniques.

## Conclusion

The present study categorizes the applications of IoT, IoT-ML, and ML-based techniques to tackle COVID-19-related problems. The main applications are monitoring, detection, identification, classification, and diagnosis. Studying, comparing, and investigating these applications requires a proper judgment about the performance and effectiveness of outputs. According to a deep consideration of the evaluation criteria, it has been investigated that the accuracy, followed by the recall and precision parameters, have owned the highest portion of the evaluation criteria employed for analyzing COVID-19 based dataset using IoT and ML-based techniques.

Most of the studies lack numerical analysis for the method performance. One of the main reasons can be the nature of the IoT technique which goes through a practical process and shows its performance in practical applications. In all the applications, IoT could successfully cope with the tasks. Such that, IoT provided a fast and efficient approach to tracking the disease spread. Most of the studies developed by ML-based techniques for handling COVID-19-based datasets provided performance criteria. According to the results section, the following points can be extracted:

- IoT provided a fast and efficient approach to tracking the disease spread.
- IoT can be employed as a real-time framework to minimize the impact of communicable diseases through early detection of cases.
- The most popular performance criteria are related to the accuracy factor.
- ML-based methods are able to be used with different types of datasets.
- CNN with SVM classifier, Genetic CNN, and pre-trained CNN followed by ResNet, provided the highest accuracy values.
- A.I. is a result-oriented technology employed for proper screening, analysis, forecasting, and tracking of current and potential future patients.

Policy-making in COVID-19 disease to examine the weaknesses and strengths and vulnerabilities of society in terms of the penetration of pathogenic viruses can be considered additional measures and future studies. On the other hand, the study of collective behaviors can also be considered as a perspective to complete studies to prevent similar social harms, reduce costs incurred, and not surprise human life. The future perspective is to employ an advanced analytic ML-based platform that supports huge-data analytics. This trend moves toward smart health interconnected with innovative technologies in the sensor industry. The future is waiting for tremendous promotion in smart health.

## Data Availability Statement

The original contributions presented in the study are included in the article/supplementary material, further inquiries can be directed to the corresponding author/s.

## Author Contributions

SB and SA designed the study. SA, AY, BP, AK, and AM wrote the paper. SB, AB, HA-R, MM, and AK edited the manuscript. SA, AY, and BP carried out all the analyses. SB, SA, AY, BP, and MM generated all figures and all tables. HA-R and AB were not involved in any analyses. All authors have read and approved the final version of the paper.

## Conflict of Interest

The authors declare that the research was conducted in the absence of any commercial or financial relationships that could be construed as a potential conflict of interest.

## Publisher's Note

All claims expressed in this article are solely those of the authors and do not necessarily represent those of their affiliated organizations, or those of the publisher, the editors and the reviewers. Any product that may be evaluated in this article, or claim that may be made by its manufacturer, is not guaranteed or endorsed by the publisher.
